# *BRCA2* carriers with male breast cancer show elevated tumour methylation

**DOI:** 10.1186/s12885-017-3632-7

**Published:** 2017-09-11

**Authors:** Siddhartha Deb, Kylie L. Gorringe, Jia-Min B. Pang, David J. Byrne, Elena A. Takano, kConFab Investigators, Alexander Dobrovic, Stephen B. Fox

**Affiliations:** 1 Molecular Pathology Research and Development Laboratory, Department of Pathology, Peter MacCallum Cancer Centre, Melbourne, VIC 3000 Australia; 2 Sir Peter MacCallum Department of Oncology, The University of Melbourne, Vic, Parkville, 3010 Australia; 3 Cancer Genomics Program, Peter MacCallum Cancer Centre, Melbourne, VIC 3000 Australia; 4 Department of Pathology, University of Melbourne, Parkville, VIC 3012 Australia; 5 Kathleen Cuningham Foundation Consortium for research into Familial Breast Cancer, Peter MacCallum Cancer Centre, Melbourne, 3000 Australia; 6 Translational Genomics and Epigenomics Laboratory, Olivia Newton-John Cancer Research Institute, Heidelberg, VIC 3084 Australia; 7 School of Cancer Medicine, La Trobe University, Bundoora, VIC 3084 Australia

**Keywords:** Male breast cancer, Familial breast cancer, Methylation, BRCA1, BRCA2, Promoter methylation

## Abstract

**Background:**

Male breast cancer (MBC) represents a poorly characterised group of tumours, the management of which is largely based on practices established for female breast cancer. However, recent studies demonstrate biological and molecular differences likely to impact on tumour behaviour and therefore patient outcome. The aim of this study was to investigate methylation of a panel of commonly methylated breast cancer genes in familial MBCs.

**Methods:**

60 tumours from 3 *BRCA1* and 25 *BRCA2* male mutation carriers and 32 males from BRCAX families were assessed for promoter methylation by methylation-sensitive high resolution melting in a panel of 10 genes (*RASSF1A*, *TWIST1*, *APC*, *WIF1*, *MAL*, *RARβ*, *CDH1*, *RUNX3*, *FOXC1* and *GSTP1*). An average methylation index (AMI) was calculated for each case comprising the average of the methylation of the 10 genes tested as an indicator of overall tumour promoter region methylation. Promoter hypermethylation and AMI were correlated with *BRCA* carrier mutation status and clinicopathological parameters including tumour stage, grade, histological subtype and disease specific survival.

**Results:**

Tumours arising in *BRCA2* mutation carriers showed significantly higher methylation of candidate genes, than those arising in non-*BRCA2* familial MBCs (average AMI 23.6 vs 16.6, *p* = 0.01, 45% of genes hypermethylated vs 34%, *p* < 0.01). *RARβ* methylation and AMI-high status were significantly associated with tumour size (*p* = 0.01 and *p* = 0.02 respectively), *RUNX3* methylation with invasive carcinoma of no special type (94% vs 69%, *p* = 0.046) and *RASSF1A* methylation with coexistence of high grade ductal carcinoma in situ (33% vs 6%, *p* = 0.02). Cluster analysis showed MBCs arising in *BRCA2* mutation carriers were characterised by *RASSF1A, WIF1*, *RARβ* and *GTSP1* methylation (*p* = 0.02) whereas methylation in BRCAX tumours showed no clear clustering to particular genes. *TWIST1* methylation (*p* = 0.001) and AMI (*p* = 0.01) were prognostic for disease specific survival.

**Conclusions:**

Increased methylation defines a subset of familial MBC and with AMI may be a useful prognostic marker. Methylation might be predictive of response to novel therapeutics that are currently under investigation in other cancer types.

**Electronic supplementary material:**

The online version of this article (10.1186/s12885-017-3632-7) contains supplementary material, which is available to authorized users.

## Background

Male breast cancer (MBC) is a poorly studied disease. Indeed, MBC accounts for ~1% of all breast cancers but it contributes to a higher proportion of breast cancer-related deaths [1, 2]. As a significant proportion of MBCs arise within breast/ovarian families, the majority of MBC research has focused on cancer predisposition. However, differences in genotype-phenotype between female and male breast cancers suggest that MBCs have alternate and novel drivers [3–5].

It is now well recognised that aberrant modification of gene expression by promoter methylation is often pathogenic and not an inconsequential contributor to oncogenesis: indeed epigenomic changes are often more commonly observed than gene mutations and chromosomal instability in many cancers [6]. In cancer, aberrant methylation is frequently seen within CpG islands in promoter regions often resulting in transcriptional silencing [7] often occurring early in cancer development. From a clinical perspective, gene methylation may not only contribute to the biological understanding of cancer subsets, but may also be utilised in screening, staging and monitoring of disease activity, as methylation is stable in formalin-fixed paraffin-embedded pathology material and in plasma. Methylated genes may also be attractive treatment targets in MBC using therapies in trials in other tumour types [8].

To date only three MBC studies, composed of a total of 182 male breast cancers, have evaluated methylation in MBCs, which showed that promoter gene methylation in MBC, as compared to normal male breast tissue, is a common event and associated with a more aggressive phenotype [9–11]. However, the methodologies used are prone to give false positive results and/or are non-quantitative. To address the paucity of data we have performed methylation profiling in a well-characterised series of MBC. Our aims were to 1) determine the frequency and level of methylation of important breast cancer genes in a large cohort of familial MBCs, 2) identify clinicopathological associations, including patient outcome, that may define a biological effect of gene methylation and 3) identify and characterise potential molecular subgroups defined by their methylation patterns with clinicopathological correlation.

## Methods

### Patient samples

Primary male breast cancers examined in this study were obtained from the Kathleen Cunningham Foundation Consortium (kConFab) breast/ovarian familial cancer repository (Table 1). Cases are accepted into the registry based on a strong family history of breast and ovarian cancer with criteria for admission to the kConFab study as outlined previously [12], with all participants providing informed consent to participate in research studies. Patients were from Australia and New Zealand and diagnosed between 1980 and 2009.

**Table 1:** Clinicopathological description of male breast cancers in this study

Feature		
Age (years)	Median = 62.5	Range: 30–85
Mutation carrier status		
*BRCA1*	3	5.0%
*BRCA2*	25	41.7%
*BRCAX*	32	53.3%
Size (mm)	Median = 17	Range: 2–50
Histological subtype		
Invasive carcinoma - no special type (IC-NST)	46	76.7%
Invasive papillary carcinoma	8	13.3%
IC-NST with areas of micropapillary	4	6.7%
Invasive lobular carcinoma	2	3.3%
Grade		
1	2	3.3%
2	30	50.0%
3	28	46.7%
DCIS		
Present	41	68.3%
Absent	15	25.0%
Unknown	4	6.7%
Nodal Status		
N0	28	46.7%
N1	20	33.3%
Nx	12	20.0%
Paget’s Disease		
Present	8	13.3%
Absent	44	73.3%
Unknown	8	13.3%
ER status (Allred score)		
Negative (0–4/8)	2	3.3%
Positive (5–8/8)	58	96.7%
PgR status (allred score)		
Negative (0–4/8)	8	13.3%
Positive (5–8/8)	52	86.7%
HER2 (SISH)		
Amplified	5	8.3%
Non-amplified	55	91.7%
Phenotype		
Luminal	54	90.0%
HER2	5	8.3%
Basal	1	1.7%

The flow of patients through the study was according to the REMARK criteria outlined in Additional file 1 [13]. Of the 118 cases within the kConFab registry, 58 cases were excluded due to unavailability of tissue. Sixty cases had sufficient material at an appropriate DNA concentration for methylation testing as outlined below. These cases belonged to three groups: 3 MBCs that arose in *BRCA1* mutation carriers, 25 that arose in *BRCA2* mutation carriers and 32 that occurred in males from BRCAX families (i.e. where an underlying germline mutation had not been identified).

Clinical parameters, including disease specific survival (DSS) were obtained from referring clinical centres, kConFab questionnaires and state death registries [14, 15]. Information on pedigrees, mutational status and testing were available from the kConFab central registry. Histological classification was based on criteria set by the World Health Organisation 2012 [16] and all slides and pathological records from all cases were reviewed centrally. Immunohistochemistry for estrogen receptor (ERα), progesterone receptor (PgR), basal markers (cytokeratin 5 (CK5), EGFR) and HER2 silver in-situ hybridisation (SISH) was performed as previously reported [4]. Stratification of intrinsic phenotypes was based on Nielsen et al. [17], and placed into luminal (ERα/PgR positive, HER2 negative, CK5 and/or EGFR negative), basal (ER α/PgR and HER2 negative; CK5 and/or EGFR positive), HER2 (HER2 positive) and null/negative (HER2, ERα, PgR, CK5 and EGFR negative) phenotypes. Permission to access the kConFab samples and data was granted by the kConFab Executive Committee (Project #115/07–17). This work was carried out with approval from the Peter MacCallum Cancer Centre Ethics Committee (Project No: 11/61).

### Germline *BRCA1*/*2* testing

Mutation testing for *BRCA1* and *BRCA2* mutations was performed as previously reported [18, 19]. Once the family mutation had been identified, all pathogenic (including splice site) variants of *BRCA1* and *BRCA2* were genotyped by kConFab in all available family members’ DNA.

### DNA extraction

Genomic DNA was extracted from formalin-fixed, paraffin embedded (FFPE) samples. A 3 μM haematoxylin and eosin (H&E) stained slide was cut from FFPE blocks and stained to identify for tumour enriched areas showing >80% tumour purity. From the relevant area on the FFPE block, one to two 2 mm punch biopsy cores were taken. The cores were then dewaxed and hydrated through a decreasing alcohol series. Genomic DNA was then extracted using the DNeasy Tissue kit (Qiagen, Hilden, Germany) following proteinase K digestion at 56 °C for 3 days.

### Bisulfite modification

Genomic DNA (600 ng) was bisulfite modified using the MethylEasy™ Xceed kit (Genetic Signatures, North Ryde, Australia) according to the manufacturer’s instructions. The bisulfite modified DNA was eluted into 50 μL of EB buffer. CpGenome™ Universal Methylated DNA (Chemicon/Millipore, Billerica, MA) and whole-genome amplified DNA [20] were used as the fully methylated and unmethylated controls, respectively. DNA methylation standards (10, 25 and 50%) were made by mixing the fully methylated control with the unmethylated DNA control.

### Methylation-sensitive high resolution melting (MS-HRM)

Methylation screening was performed using MS-HRM to quantitate methylation in bisulfite-modified samples according to the sequence-dependent thermostability in which the level and presence of homogenous and heterogeneous methylation can be detected [21, 22]. MS-HRM primers were specifically designed to generate short amplicons enabling use in formalin-fixed paraffin embedded (FFPE) samples and are summarised in Additional file 2.

**Fig. 1:**
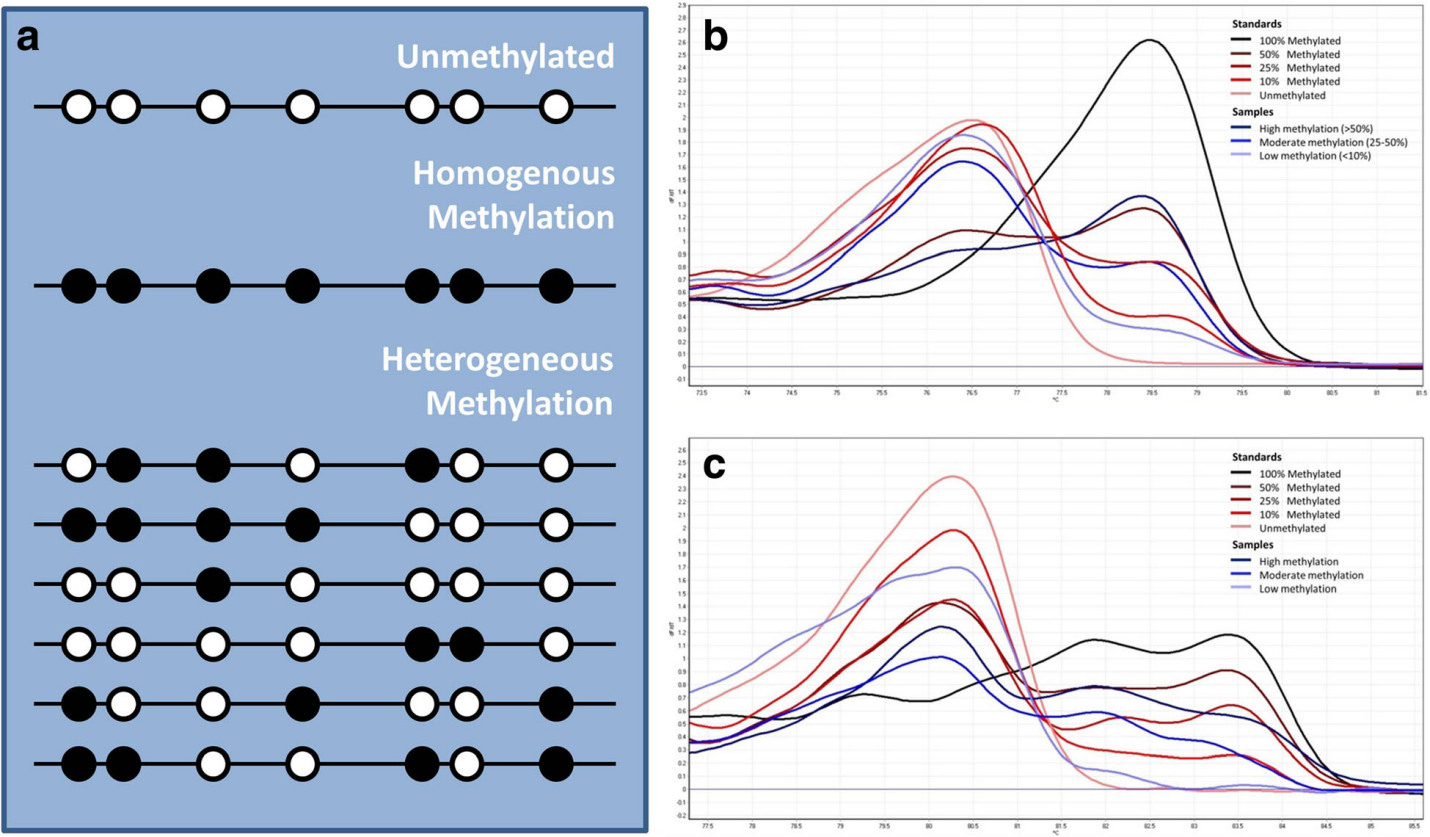
**a** Schematic representation of an unmethylated sample, homogenously methylated sample and heterogeneously methylated sample (circles represent CpG islands with white indicating unmethylated and black indicating methylated sites), **b** quantitation of homogenous methylation (*RARβ*), **c** quantitation of heterogeneous methylation (*RUNX3*)

PCR amplification and HRM analysis were performed on the Rotor-Gene 6000 (Corbett, Sydney). Samples were run in duplicate. Conditions for each gene are described in Additional file 2. The reaction was performed using a final volume of 20 μL and the mixture consisted of 1 × PCR buffer (Qiagen, Hilden, Germany), 2.5–4.0 mmol/L of MgCl_2_, 200 μmol/L of each dNTP, forward and reverse primers, 5 μmol/L of SYTO9 intercalating dye (Invitrogen, Carlsbad, CA), 0.5 U of HotStarTaq DNA polymerase (Qiagen, Hilden, Germany) and 10 ng of bisulfite modified DNA. The methylation level of each DNA sample was determined visually by comparing it against the standard curves. Heterogeneous DNA methylation was defined by melting profiles that did not directly conform to any of the methylation controls due to the formation of heteroduplexes between closely but not identically related single complementary DNA strands. Complexes that complete melting slightly after the unmethylated controls were indicative of low levels of DNA methylation. In contrast, complexes with a late melting profile typically contained more heavily methylated epialleles (Fig. 1).

### Methylation scoring

A cut-off of 10% methylation was used to primarily exclude low level methylation of uncertain biological significance. The remaining samples were further grouped into moderate methylation (10–50% fully methylated, or moderate heterogenous methylation) and high methylation (>50% fully methylated, or high-level heterogenous methylation) (Fig. 1). Positive methylation (hypermethylation) for each gene was thus considered when duplicate samples showed >10% or moderate to high heterogeneous methylation The samples were also given a percentage methylation for each gene by comparing the methylation to the curves of the standard, which was then averaged across all the genes to give a average methylation index (AMI) scored between 0 and 100% for each tumour sample [23]. The AMI measurement is based on the cumulative methylation index [24], which is the sum of the percentages of methylation of the individual genes, but corrects for the number of genes tested*.* Using the AMI scores, groups were dichotomised into low and high based on the median AMI as a cut-off point. This analysis does not make assumptions as to the effect of any particular level of methylation.

### Cluster analysis

Unsupervised complete linkage clustering was performed with Euclidean metric distance. Unsupervised hierarchical cluster analysis of methylation at each gene was used to detect possible distinct molecular signatures. Analysis was performed using Cluster and Tree View software written by Michael Eisen (Stanford University) as previously published [25–27].

### Statistical analysis

Comparison of groups was made with using Mann-Whitney U for non-parametric continuous distributions and Fisher’s exact test for threshold data. Kaplan-Meier survival curves were plotted using breast cancer related death as the endpoint and compared using a log rank test. Pearson’s correlation coefficient was measured for the cluster analysis. Analysis was performed with GraphPad Prism 5 software (GraphPad Prism version 5.04 for Windows, GraphPad Software, La Jolla California USA). A two-tailed *P*-value test was used in all analyses and a *p*-value or less than 0.05 was considered statistically significant.

## Results

### Methylation analysis of MBCs finds associations with genotype and clinico-pathological characteristics

We performed methylation analysis on 60 MBC (25 *BRCA2*, 3 *BRCA1* and 32 BRCAX), whose clinical and pathological features are summarised in Table 1. The features of these cases are consistent with familial male breast cancers in the literature [28], primarily being invasive carcinomas of no special type (76%), ER and PR positive (97% and 87% respectively) and HER2 unamplified (92%). Fifty four (90%), five (8%) and one (2%) tumour(s) were luminal, HER2 and basal phenotypes respectively.

We selected 10 genes for analysis based on their frequency of methylation and/or association with prognosis in previous studies of breast cancer, as follows. Methylation of *GSTP1 and RASSF1A* is common in MBC [10, 11]. Methylation of *WIF1*, *TWIST*, *FOXC1*, *APC, RARb* and *MAL* have also been associated with patient outcome in FBC [29–33]. *CDH1*, *RARB* and *RUNX3* are frequently methylated in 22–72% [34–36], 20–45% [35, 37, 38] and 50–90% of FBC respectively [39, 40].

*GSTP1* was the most commonly methylated gene (82%), followed by *RASSF1A* (68%), with both showing a pattern of predominantly high level methylation (Table 2). Other genes were more varied: *RARβ*, *APC* and *RUNX3* had moderate levels of methylation, while heterogeneous methylation was observed in *TWIST1*, *MAL* and *WIF1*, with a mix of moderate and high heterogeneous methylation. Only low level methylation was observed in *CDH1* with no cases showing hypermethylation. There were no statistically significant associations of specific gene methylation with patient genotype, however, there were trends for higher methylation frequency of *RARβ* (44% vs 20%, *p* = 0.08) and *TWIST1* (52% vs 26%, *p* = 0.06) in *BRCA2* carriers. Overall, the *BRCA2* group also showed a higher rate of gene hypermethylation (45% vs 34%, *p* < 0.01) in our target suppressor gene panel than the other groups.

**Table 2:** Percentage of cases with hypermethylation

	*GSTP1*	*RASSF1A*	*MAL*	*TWIST*	*RUNX3*	*RARβ*	*APC*	*FOXC1*	*CDH1*	*WIF1*	TOTAL HYPERMETHYLATED GENES	AMI (mean)
*BRCA1* (*n* = 3)	2 (66%)	1 (33%)	1 (33%)	0	1 (33%)	1 (33%)	1 (33%)	1 (33%)	0	1 (33%)	9 (30%)	13.4
*BRCA2* (*n* = 25)	22 (88%)	20 (80%)	14 (56%)	13 (52%)	8 (32%)	11 (44%)	8 (32%)	6 (24%)	0	11 (44%)	113 (45%)	23.6
*BRCAX* (*n* = 32)	25 (78%)	20 (63%)	12 (38%)	9 (28%)	9 (28%)	6 (19%)	7 (22%)	8 (25%)	0	14 (56%)	110 (34%)	17.0
All (*n* = 60)	49 (82%)	41 (68%)	27 (45%)	22 (37%)	18 (30%)	18 (30%)	16 (27%)	15 (25%)	0	26 (43%)	232 (39%)	14.0
				*p* = 0.06		*p* = 0.08					*P* < 0.01	*p* = 0.01

We examined the association of specific gene methylation with patient and tumour characteristics (Table 3). *APC* hypermethylation was significantly associated with older age (69.1 years vs 60.4 years, *p* = 0.01, Table 2) whereas *MAL* hypermethylation was significantly inversely associated with age (59.1 years vs 65.7 years, *p* = 0.04). Significantly larger tumour size was noted for cases with *RARβ* hypermethylation (median 22.3 mm vs 16.5 mm; *p* = 0.01). *RARβ* hypermethylation was also associated with a higher percentage of Paget’s disease (31% vs 8%, *p* = 0.04). *RUNX3* hypermethylation was associated with increased frequency of IC-NST histological type (94% vs 69%, *p* = 0.046) and *RASSF1A* hypermethylation associated with the coexistence of high grade DCIS (33% vs 6% (*p* = 0.02).

**Table 3:** Correlation of hypermethylation with clinicopathological variables (associations approaching significance, *p* < 0.05 in bold)

	*GSTP1*		*RASSF1A*		*MAL*		*RUNX3*		*RARβ*		*APC*	*FOXC1*			AMI (median)	
Hypermethylation	+	-	+	-	+	-	+	-	+	-	+	-	+	-	>	<
Age (years)					59.1	65.7			67.2	60.9	69.1	60.4				
*p*-value						**0.04**				0.07		**0.01**				
Tumour size (mm)									22.3	16.5	21.4	17.1			20.8	15.8
*p*-value										**0.01**		0.08				**0.02**
IC-NST Histology							94%	69%								
*p*-value								**0.046**								
Grade 3	51%	18%														
*p*-value		0.09														
Paget’s Disease									31%	8%						
*p*-value										**0.04**						
DCIS present			33%	6%												
*p*-value				**0.02**												
Lymphovascular invasion	49%	18%									20%	51%	53%	40%		
*p*-value		0.09										0.07		0.09		
Perineural invasion									63%	36%						
*p*-value										0.07						
Node positive			52%	24%					62%	34%						
*p*-value				0.08						0.07						
HER2 positive															13%	0
*p*-value																0.11

**Fig. 2:**
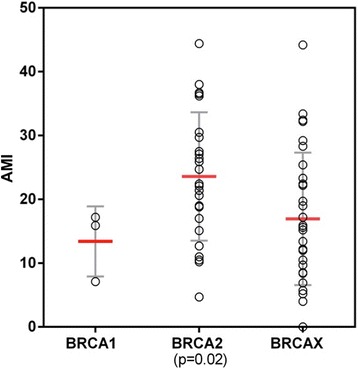
Average methylation index (AMI) for samples stratified by BRCA status (Central bar – median, error bars = 1 standard deviation)

High overall levels of methylation have been associated with aggressive tumour features such as mitotic count, grade and poor patient outcome in MBC [10] and FBC [30, 41]. Therefore, we calculated a measure of overall methylation for each sample, the AMI. There was a significant increase in AMI in *BRCA2* mutation carriers compared with other MBCs (23.6 vs 16.6, *p* = 0.01, Fig. 2). In addition, the AMI was positively correlated with tumour size (median 22.4 mm vs 15.4 mm, *p* = 0.02).

### Cluster analysis identifies subgroups of MBC

**Fig. 3:**
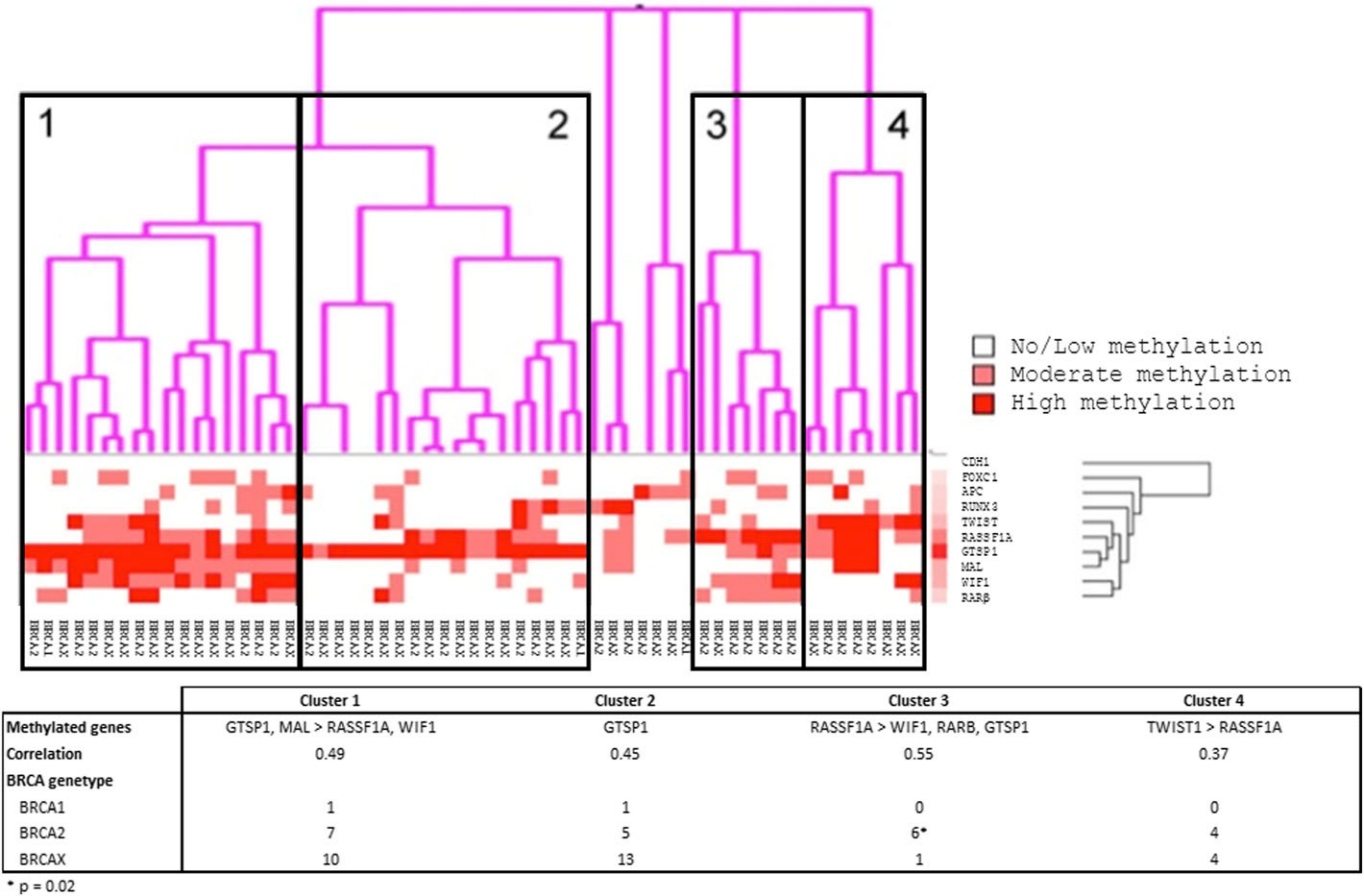
Unsupervised cluster analysis of methylation amongst male breast cancers (gradation is seen in the shading between white (no methylation) and red (high methylation))

In order to evaluate whether methylation profiles could discover novel subgroups in MBC, as has been seen for FBC [42, 43] and colorectal cancer [44], we performed an unsupervised clustering analysis. Four main clusters with at least 7 samples in each group were identified (Fig. 3). MBCs arising in *BRCA2* carriers showed a significantly greater frequency (6/7 vs 19/53, *p* = 0.02) of Cluster 3 membership (characterised by *RASSF1A, WIF1, GSTP1* and *RARβ* methylation). No other clinicopathological association or prognostic differences were seen between the clusters.

Analysis of methylation patterns within the *BRCA2* subgroup of tumours showed two clusters with correlation coefficients >0.8) (Additional file 3). Cluster A contained 12 tumours and was characterised by high *GSTP1* methylation and *MAL* methylation and relatively lower *RASSF1A* methylation. Cluster B contained 8 tumours and showed primarily high *RASSF1A* methylation. Cluster A tumours showed an earlier age at diagnosis than other *BRCA2* tumours. Other variables did not align to one or the other cluster. Analysis of BRCAX tumours by cluster analysis showed only very small clusters of 6 or less patients with a correlation coefficient above 0.8 (Additional file 3).

### A high average methylation index and *TWIST1* hypermethylation associated with worse disease specific survival

**Fig. 4:**
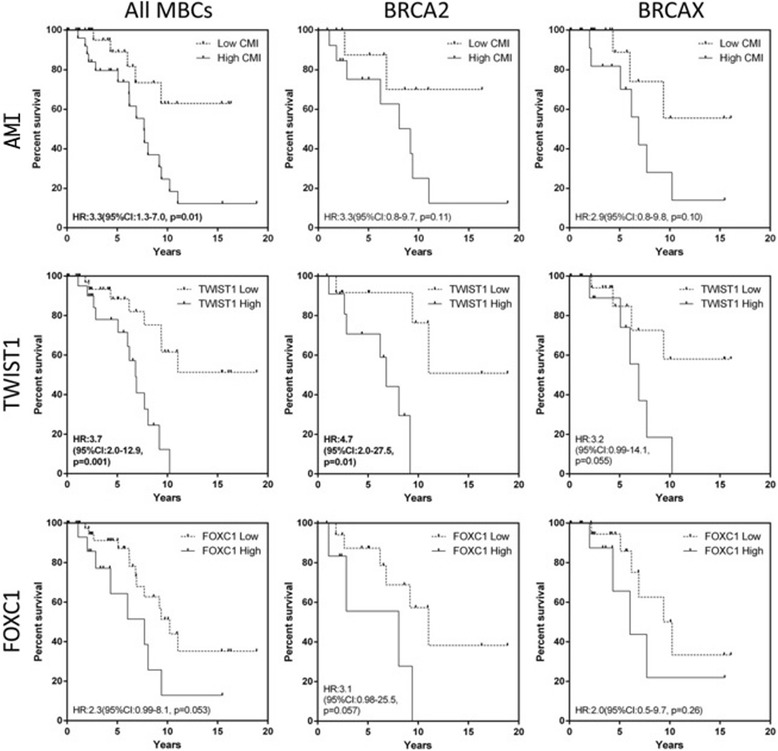
Disease specific survival of average methylation index (AMI), *TWIST1* and *FOXC1* in all male breast cancers and within the *BRCA2* and BRCAX subgroups

Both a high AMI (HR:3.3, 95% CI:1.3–7.0, *p* = 0.01) and hypermethylation of *TWIST1* (HR:3.7, 95% CI:2.0–12.9, *p* = 0.001) were adverse features for disease specific survival (Fig. 4) with *TWIST1* methylation (HR:4.7, 95% CI:2.0–27.5, *p* = 0.01) also being associated with a significantly shorter survival in the *BRCA2* MBC subgroup. Because *BRCA2* tumours have higher methylation overall and also worse survival than other MBC cohorts [45, 46], we also evaluated survival within the *BRCA2* carriers, and observed a trend towards worse outcome with higher AMI in this sub-group (HR:3.3, 95% CI: 0.8–9.7, *p* = 0.1). Hypermethylation of *FOXC1* (HR:2.3, 95% CI:0.99–8.1, *p* = 0.053) showed a strong trend towards worse DSS; hypermethylation of other genes showed no prognostic information. No significant association with progression-free survival was detected for any gene or AMI. Multivariate analysis was not performed due to inadequate numbers of cases.

## Discussion

Aberrant methylation of promoter regions of tumour suppressor genes has been shown to be a frequent mechanism of gene silencing in most cancers, including breast cancers [47–49]. In many instances, this is observed in adjacent normal tissues or in pre-invasive lesions [50]. Perhaps best seen in colorectal cancer [51], subsets may demonstrate methylation patterns with clinical relevance.

We have used methylation sensitive high-resolution melting analysis of methylation as it has been demonstrated to be highly sensitive, robust and effective in evaluating FFPE tissue, able to differentiate and semi-quantitate homogenous and heterogeneous methylation [22, 52]. This current comparative study is the largest to examine methylation using a robust technology of well characterised and acknowledged tumour suppressor genes shown to be methylated and important in the pathogenesis FBC, in a clinically well annotated cohort of familial male breast cancers with known mutation status. We have identified frequent promoter hypermethylation (≥30%) in *GSTP1*, *RASSF1A*, *MAL*, *TWIST*, *RUNX3*, and *RARβ*, and identified significant associations with clinico-pathological features in five of the genes assayed. One caveat to some of these associations is that the small sample size and their level of statistical significance close to the *p* < 0.05 threshold may mean that false positive results are included due to the multiple tests performed.

Currently there are only three published methylation studies in a total of 182 male breast cancers. Of the genes we investigated only methylation at *GSTP1*, *RARβ* and *RASSF1A* have been individually assessed, The largest study by Kornegoor et al. [10] examined candidate methylation of 25 genes in 108 MBCs by methylation specific multiplex ligation dependent probe amplification (MS-MLPA), detecting methylation in *RARβ* (5%) and *GSTP1* (44%), somewhat lower than our results. This study did not segregate MBC into sporadic and familial groups, which have been shown to contain distinct geno-phenotypic characteristics and may explain the difference in frequency observed. The second study by Pinto et al. [11] evaluated *RASSF1A* (76%) and *RARβ* (8%) in 27 familial MBCs using quantitative methyl-specific PCR. The lower frequency of *RARβ* hypermethylation observed may be explained by the lower proportion of *BRCA2* cases included (3/27 compared to 25/60 in our cohort). Consistent with this possibility we observed a trend for *RARβ* methylation to be higher in *BRCA2* cases. Finally, Johanssen et al. [9] performed genome-wide methylation profiling in 47 MBCs, and identified two clusters of cases; unfortunately germline mutation status was only available for 8 cases.

One of the most striking findings in this study is the high frequency of *GSTP1* methylation (82%), which has not been noted before. *GSTP1* encodes for glutathionine S transferase P [53] and may be a critical gene in the development of familial MBCs. Very high levels of *GSTP1* methylation are also seen in prostate cancer, which is another male cancer that can be associated with *BRCA2* mutation [54, 55]. We noted high levels of *GSTP1* methylation in both *BRCA2* (88%) and BRCAX tumours (78%), well above that noted by Kornegoor et al. (44%) and that reported in FBCs (generally <60%) [56, 57]. The reason for this result is unlikely to be assay related, as using the same methodology we have shown similar levels of methylation in FBC to that reported in the literature. There are two other possibilities. Firstly, *GSTP1* methylation may be ERβ mediated as studies of prostate cancer lines show that the ERβ/eNOS complex causes *GSTP1* repression by local chromatin remodelling following recruitment to estrogen responsive elements [58]. Secondly, *GSTP1* functions as a caretaker gene [53, 58, 59] with its loss resulting in increased oxidative DNA damage and mutagenesis, thus, in *BRCA2* deficient cancers already sensitive to oxidative stress [60], any loss of *GSTP1* may have a more pronounced effect and be integral in tumour development.

We also noted overall methylation differences between the *BRCA2* and BRCAX subgroups further supporting previous studies showing a possible *BRCA2* MBC subset. In female *BRCA2* carriers, promoter hypermethylation has also been shown to be elevated compared to non-familial and *BRCA1* carriers [49, 61]. Methylation profiling of FBC was able to discriminate *BRCA1*, *BRCA2* and two subsets of BRCAX tumours [61]. This study is the first to report on methylation of male breast cancers arising in *BRCA1* mutation carriers. These tumours are rare, and while we only have three cases within our cohort, this is a novel group. We were unable to see a significant correlation between gene hypermethylation and *BRCA1* status but did observe the lowest levels of methylation of all the groups, mirroring the findings seen in *BRCA1* associated female breast cancer. Further investigation of this rare subgroup is warranted.

This high level of methylation could potentially be used for screening in *BRCA2* male carriers as methylation is not seen in normal tissues, serum or plasma of normal individuals but can be detected in blood. *GSTP1* may be the prime candidate as studies evaluating its use as a biomarker for prostate cancer are well advanced.

To aid the above possible screening strategies we have developed an index of methylation (AMI) to investigate the quanta of methylation. We observed that AMI correlated with larger tumour size and shorter disease specific survival suggesting that either a stochastic accumulation of methylation and/or a methylator phenotype leads to a more aggressive tumour, as observed in the study of Kornegoor et al. [10]. Similarly, Johansson et al. [9] found that a highly methylated MBC subgroup was more proliferative and showed a trend towards worse patient outcome. In sporadic FBC conflicting results regarding methylation and survival have been found, with higher methylation subgroups showing either improved prognosis [43] or poor overall survival [62]. These differences are perhaps explained by the influence of the intrinsic subtypes, which show distinct methylation patterns and patient outcome [49]. The association between multi-gene hypermethylation and outcome in familial FBC does not appear to have been evaluated. Notably, in our cohort a high AMI maintained a trend towards prognostic significance in *BRCA2* tumours further suggesting that as above, methylation has particular biological importance in this subset of tumours.

## Conclusions

We have shown that tumour promoter methylation within our target suppressor gene panel is commonly observed in familial and particularly *BRCA2* male breast cancers suggesting aberrant hypermethylation may be a significant driver in MBCs carrying prognostic information. In addition, the presence of specific methylation patterns particular to MBC subtypes such as *BRCA2* carriers further supports emerging evidence suggesting the presence of unique and distinct MBC subsets that differ from other MBC subgroups and from FBC.

## Additional files

Additional file 1: Table S1. REMARK patient flow through study (XLSX 34 kb)

Additional file 2: Table S2. Methylation specific high resolution melting condition and primers (XLSX 36 kb)

Additional file 3: Figure S1. a) BRCA2 subgroup cluster analysis, b) BRCAX subgroup cluster analysis, c) Numbers and sizes of clusters within BRCA2 and BRCAX subgroups using various correlation coefficient cut-offs (listed on the x-axis), d) age of diagnosis of patient within Cluster A, B and other BRCA2 tumours (DOCX 234 kb)

## Abbreviations

AMI: Average methylation index
CK5: Cytokeratin 5
DCIS: Ductal carcinoma in situ
DSS: Disease specific survival
ERα: Estrogen receptor
FBC: Female breast cancer
FFPE: Formalin-fixed, paraffin embedded
H&E: Haematoxylin and eosin
IC-NST: Invasive carcinomas of no special type
kConFab: Kathleen Cuningham Foundation Consortium
MBC: Male breast cancer
MS-HRM: Methylation-sensitive high resolution melting
MS-MLPA: Methylation-specific multiplex ligation dependent probe amplification
PgR: Progesterone receptor
QMSP: Quantitative methyl-specific PCR
