# Supplementary material for: BRCA2 carriers with male breast cancer show elevated tumour methylation
Source: BMC Cancer. 2017 Sep 11;17:641. doi: 10.1186/s12885-017-3632-7 (PMC5594583; doi:10.1186/s12885-017-3632-7)

**Supplementary figure 1:** a) BRCA2 subgroup cluster analysis, b) BRCAX subgroup cluster analysis, c) Numbers and sizes of clusters within BRCA2 and BRCAX subgroups using various correlation coefficient cut-offs (listed on the x-axis), d) age of diagnosis of patient within Cluster A, B and other BRCA2 tumours.


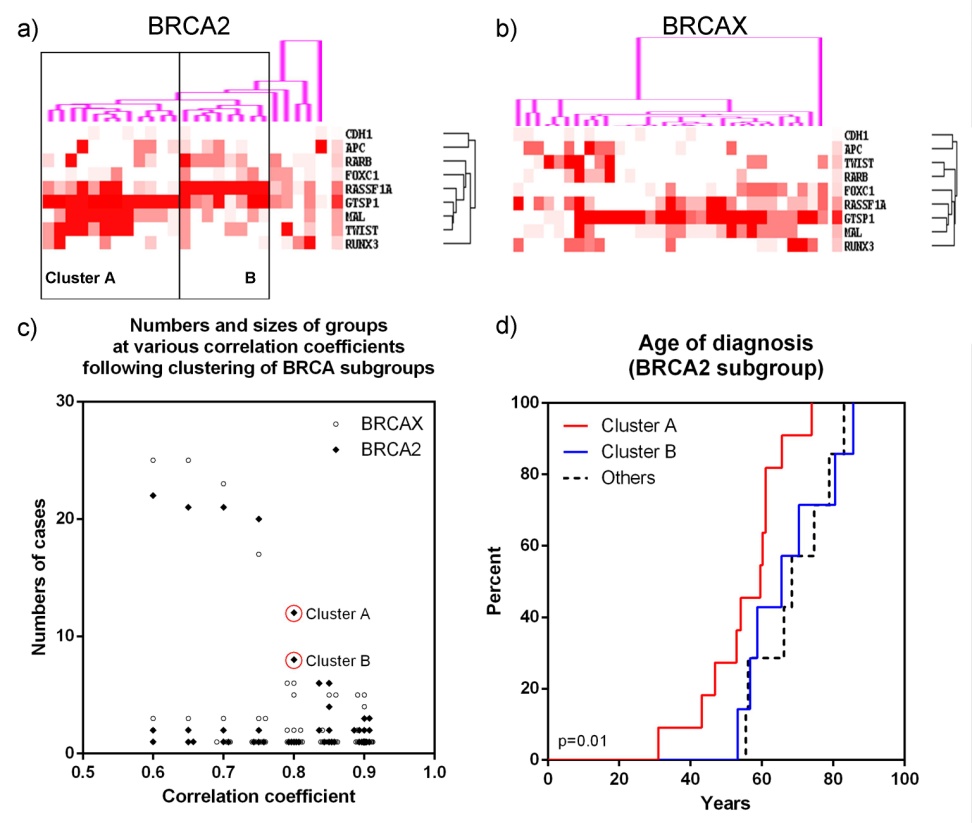

Supplement: Supplementary file 3 — a) BRCA2 subgroup cluster analysis, b) BRCAX subgroup cluster analysis, c) Numbers and sizes of clusters within BRCA2 and BRCAX subgroups using various correlation coefficient cut-offs (listed on the x-axis), d) age of diagnosis of patient within Cluster A, B and other BRCA2 tumours (DOCX 234 kb) [file 12885_2017_3632_MOESM3_ESM.docx]
